# Selective Use of Peri-Operative Steroids in Pituitary Tumor Surgery: Escape from Dogma

**DOI:** 10.3389/fendo.2013.00030

**Published:** 2013-03-18

**Authors:** Jacqueline Regan, Joseph Watson

**Affiliations:** ^1^Department of Neurosciences, Inova Health SystemsFalls Church, VA, USA; ^2^Department of Neurosciences, Inova Fairfax Hospital, Virginia Commonwealth UniversityFalls Church, VA, USA

**Keywords:** pituitary adenoma, cortisol, Rathke’s cyst, craniopharyngioma, stress-dose steroids, panhypopituitary

## Abstract

**Objective:** Traditional neurosurgical practice calls for administration of peri-operative stress-dose steroids for sellar-suprasellar masses undergoing operative treatment. This practice is considered critical to prevent peri-operative complications associated with hypoadrenalism, such as hypotension and circulatory collapse. However, stress-dose steroids complicate the management of these patients. It has been our routine practice to use stress steroids during surgery only if the patient has clinical or biochemical evidence of hypocortisolism pre-operatively. We wanted to be certain that this practice was safe.

**Methods:** We present our retrospective analysis from a consecutive series of 114 operations in 109 patients with sellar and/or suprasellar tumors, the majority of whom were managed without empirical stress-dose steroid coverage. Only patients who were hypoadrenal pre-operatively or who had suffered apoplexy were given stress-dose coverage during surgery. We screened for biochemical evidence of hypoadrenalism as a result of surgery by measuring immediate post-operative AM serum cortisol levels.

**Results:** There were no adverse events related to the selective use of cortisol replacement in this patient population.

**Conclusion:** Our experience demonstrates that selective use of corticosteroid replacement is safe; it simplifies the management of the patients, and has advantages over empiric “dogmatic” steroid coverage.

## Introduction

To prevent peri-operative complications associated with hypoadrenalism, such as hypotension and circulatory collapse, administration of peri-operative stress-dose glucocorticoids is traditional in neurosurgical practice for sellar-suprasellar masses undergoing operative treatment (Yeh and Chen, 1997). However, while caring for post-operative Cushing’s disease patients who were profoundly hypocortisolemic, we observed that hypotension, other than postural hypotension, was exceedingly rare. In fact, although the patients suffered from general malaise and suffered anorexia, none were in danger of death from cardiovascular collapse. Based on this observation and supporting literature (Inder and Hunt, 2002; Wentworth et al., 2008) we have abandoned the routine use of peri-operative steroids for pituitary surgery for the last 10 years. The policy proved safe clinically, but we wanted to analyze these data to be sure that we were not being biased in our assessment. Therefore, we instituted and maintained a database of our pituitary patients, specifically examining the pre- and post-operative morning (AM) cortisol levels and recording any peri-operative adverse events.

## Patients and Methods

This study was approved by the local Institution Review Board. From November, 2007 through February 2012, all patients (*n* = 114) undergoing pituitary surgery at a single institution by a single neurosurgeon (JCW) had the following information concurrently recorded in a password secure database: age, surgery type, date, and intraoperative complications such as CSF leak; endocrine assessment including pre-operative AM cortisol and post-operative cortisol; steroid replacement use; pathology; and any adverse outcome (such as: infection, bleeding requiring transfusion, symptomatic hypotension, unexpected neurological outcome, diabetes insipidus (DI), hyper or hyponatremia, readmission within 90 days, cerebrospinal fluid leak requiring treatment, any event requiring intensive care unit admission, or death). Pre and post-op cortisol levels were measured by obtaining approximately 3.0 ml serum in a SST transport container, refrigerated and centrifuged. The Abbott ARCHITECT™ cortisol chemiluminescent micro-particle immunoassay (CMIA) was used to acquire μg/dl serum AM cortisol. Patients with a low pre-operative serum AM cortisol level (<4 μg/dl) were offered stress-dose steroid coverage peri-operatively (50 mg hydrocortisone IV every 6 h for 36 h and then oral replacement). Post-operative AM cortisol levels were not obtained in patients receiving stress-dose coverage or in Cushing’s patients and thus these patients were not included in the study.

**Figure 1:**
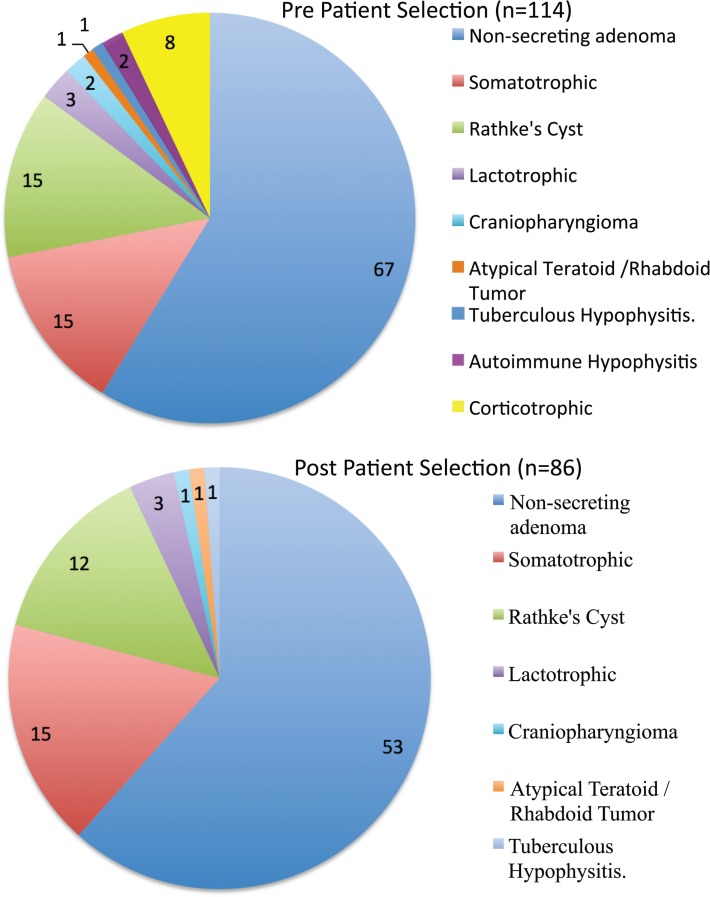
**Pre- and Post-patient selection tumor pathologies**.

The average patient age was 52.4 ± 12.0 years, with a male:female ratio of 6:5. There were only three pediatric patients. The series was dominated, as expected, by adenomas (82 macroadenomas and 11 microadenomas). The single most common surgical pathology in this series was a non-secreting macroadenoma (67/85 = 78.82%). There were 18 patients with hypersecretion syndromes (15 acromegalic, and 3 hyperprolactinemic). There were 15 Rathke’s cyst patients, two craniopharyngiomas, one atypical teratoid/rhabdoid tumor, two autoimmune hypophysitis cases, and one case of tuberculous hypophysitis (Figure 1).

### Surgery

All patients underwent a sublabial transseptal transsphenoidal or an endoscopic transnasal approach to the sella with a wide bone opening. A subcapsular dissection was performed in all cases of adenomas, as described by Oldfield and Vortmeyer (2006). An extended transsphenoidal approach was used in six cases (two adenomas, one stalk adenoma, two craniopharyngiomas, and one Rathke’s cyst) as previously described (Pluta et al., 1999).

### Analysis of the hypothalamic-pituitary-adrenal axis

#### Pre-operative assessment

Patients had AM cortisol tested pre-operatively as an assessment of the Hypothalamic-Pituitary-Adrenal (HPA) axis. In two patients, corticotropin-releasing hormone stimulation was also used. The eight patients with Cushing’s disease were excluded, as these patients do not receive intraoperative steroid replacement due to their hypercortisolemia. Also excluded were 16 patients who were on steroid treatment prior to surgery and therefore had no pre-operative assessment.

Four patients had missing AM serum cortisol pre-op. Therefore we have pre-operative data on 86 surgical cases. Those patients with a serum cortisol of <4 μg/dl in our laboratory were considered adrenal impaired for the purpose of offering stress-dose peri-operative steroid coverage.

#### Post-operative assessment

Post-operative day 1 AM cortisol was measured to determine the integrity of the HPA axis. Again, the Cushing’s patients were excluded (*n* = 8), as were patients who were maintained on exogenous steroid replacement (*n* = 16) and the four that had missing pre-operative AM cortisol.

Eighty-five non-Cushing’s patients with pituitary pathology underwent 90 surgeries without stress-dose steroid coverage. Four patients with missing pre-operative cortisol data were excluded, leaving a total of 86 surgeries with sets of pre- and post-operative AM serum cortisol samples.

The 16 patients who received peri-operative stress steroids were mostly due to apoplexy (*n* = 13) who were already on stress steroids, two patients with panhypopituitarism and one patient with chronic obstructive pulmonary disease.

The average age of only the 86 patients included was 53.2 ± 14.7 years with a male to female ratio of 9 to 4.

### Statistical analysis

IMB SPSS 19.0 statistics software (IMP Corp., Armonk, NY, USA) was used to perform statistical analysis. Difference were analyzed using a one-sided *t*-test and a *p*-value of <0.05 was considered significant.

## Results

**Figure 2:**
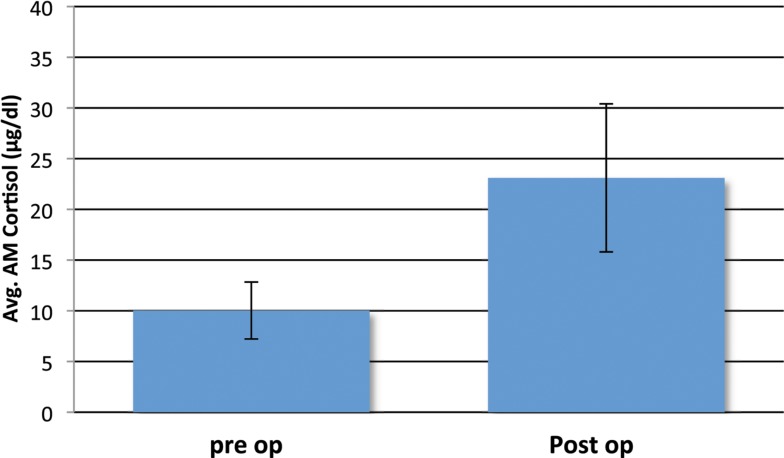
**Mean pre- and post-operative serum cortisol levels (μg/dl) in the study cohort of non-Cushing’s patients with pituitary pathology that underwent surgeries without stress-dose steroid coverage**. Four patients with missing pre-operative cortisol data were excluded. Error bars indicate standard deviation.

**Figure 3:**
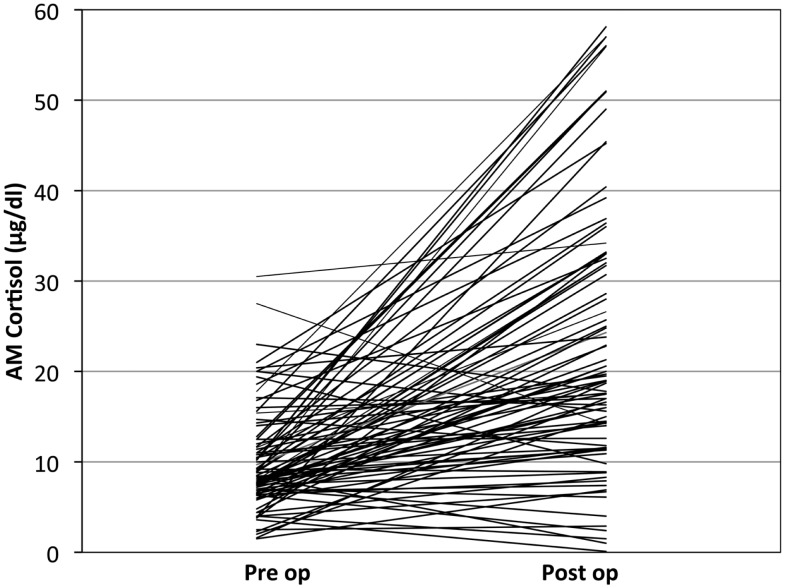
**Individual patient’s cortisol response to pituitary surgery: note that most, but not all patients operated without stress steroids show a significant rise in cortisol over baseline**.

Most post-surgical patients who were not treated with steroids were found to have a significant rise in the AM cortisol, indicative of a preserved HPA axis. By definition of the laboratories in use by our hospital, a preserved HPA axis was considered to be an AM serum cortisol of >4 μg/dl. We chose this as the base level. The mean pre-operative AM serum cortisol was 10.03 μg/dl (SD ± 5.62), and the mean post-operative AM cortisol level was 23.1 μg/dl (SD ± 14.80, one-side *t*-test; *P* < 0.001) (Figure 2). This difference can readily be seen in the individual levels (Figure 3). Although there were 12 patients who saw a decrease from pre-operative to post-operative AM cortisol levels (Figure 3) only five patients failed to achieve appropriate post-op AM cortisol levels. These patients failed to distinguish themselves clinically from those who did achieve appropriate post-op levels (i.e., not symptomatic).

There were no patients in the series developed pituitary Addison’s disease despite the five who failed to have an appropriate post-op AM cortisol of >4 μg/dl (Figure 3). Therefore screening for HPA axis impairment (solely with a pre-op AM cortisol in the vast majority of cases) was effective.

Five patients failed to mount a significant cortisol response (Figure 3). Most of these patients received stress-dose steroids post-operatively. All underwent a sublabial transseptal transsphenoidal approach to surgery. Three of these patients had relatively low pre-operative AM cortisol levels of 4, 6.3, and 8.4. These patients were diagnosed with micro prolactinoma, tuberculosis hypophysitis, and macro adenoma respectively. The remaining two patients with insignificant cortisol response had low pre-op cortisol of 3.6 and 2.5 and did not receive steroids by choice. Both were in patients with non-secreting macro adenomas. Neither of these uncovered patients had peri-operative hypotension or cardiovascular instability. One of them did have delayed anorexia and malaise and was readmitted for hyponatremia 1 week post-surgery. At 2 weeks post-surgery, this patient’s AM cortisol was normal and was asymptomatic without treatment.

### Adverse events

There were no peri-operative deaths and no cases of cardiovascular collapse, symptomatic hypotension, or cardiovascular events requiring intensive care. There were no patients who required a blood transfusion or had a new neurological deficit.

There were adverse events in our series, but none that could be explained by lack of steroid coverage. These included six patients with a CSF leak, one in a delayed fashion (1 year). One of these six patients received peri-operative steroids. There were nine cases of DI, six of which were new post-operatively. Of the six cases of post-operative DI two were adenomas, two craniopharyngiomas, and two Rathke’s cysts. There was one case of post-operative culture-negative meningitis treated with 10 days of intravenous antibiotics. This patient had presented with pituitary apoplexy and did receive peri-operative stress-dose steroids. There were five cases of symptomatic normovolemic hyponatremia (presumed SIADH): one had received steroids prior to operation and all patients recovered within 3 days of readmission for hyponatremia. One patient with acromegaly was diagnosed with a lower extremity deep venous thrombosis and pulmonary embolism 8 days post-operative. She had not received steroids.

## Discussion

Patients who had surgery for pituitary pathology did not need peri-operative stress-dose steroid coverage: there were no adverse events directly associated with performing pituitary surgery without empirical steroid coverage. One of five patients requiring readmission for hyponatremia was hypocortisolemic and it is possible that lack of glucocorticoids contributed. However, one of the five patients requiring readmission for hyponatremia was put on peri-operative stress-dose steroids. Therefore, not only was this policy safe, but it simplified patient management in all cases, as it obviated the need to treat steroid-related complications [e.g., hyperglycemia with finger stick glucose monitoring and sliding scale insulin (Al-Shoumer et al., 1995; Rajaratnam et al., 2003), and gastric ulcers with pharmacologic gastric protectors, typically proton pump inhibitors] or to wean exogenous steroids. Steroid naïveté further facilitates the endocrine assessment, as the HPA axis may be examined at any time post-surgery without obscuration by iatrogenic suppression of the axis. Additional benefits, beyond the scope of this paper, include avoiding potential steroid-related deleterious effects on wound healing, immunoresistance, behavior and sleep disturbances, and altered bone metabolism (Peacey et al., 1997; Brown and Buie, 2001; Lukert, 2006). Our policy was to treat with steroids if the patient was symptomatic from hypocortisolemic pre-operatively, such as was seen after apoplexy. Interestingly, the two patients with low pre-operative AM cortisol that were not given glucocorticoids had no dire consequences. Of further note, the one case of meningitis was in a patient who received stress-dose steroids peri-operatively.

It is important to note that it has been shown that a sodium-chloride containing fluid, like that of a saline injection, should be used as an early intervention to combat acute Addison disease crisis (Bouillon, 2006). The saline solution given to all patients during surgery could have acted as a factor preventing adrenal crisis in patients that did not receive peri-operative stress-dose steroids. This fact could make giving stress-dose steroid coverage to all patients even less necessary since patients are given saline infusion during surgery.

Medical dogma may be perpetuated by the good intentions of teachers and mentors in our hierarchical medical education system. But tradition and historically based repetition of policies may suffer from a lack of awareness of the origin of the policy or subsequent evidence-based experiences. By example, the use of empirical stress-dose glucocorticoids for pituitary surgery appears to have developed in the following way: recommendations for using high dose glucocorticoids in surgery began after two reports in the late 1950s for patients with likely adrenal suppression (Fraser et al., 1952; Lewis et al., 1953). Both reports describe the post-operative death of patients who had been on high dose cortisone prior to orthopedic surgery. The extrapolation of the experience of these cases to empirical coverage for patients with macroadenomas is ill-defined.

### Pituitary Addison’s

**Figure 4:**
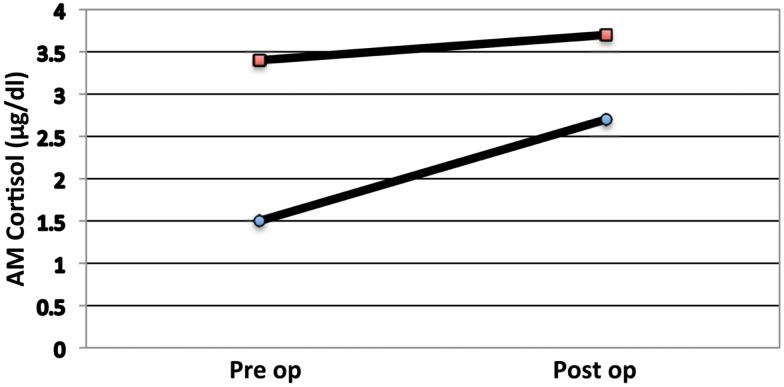
**AM serum cortisol levels of two patients with a low pre-op cortisol that, by choice, did not receive steroids**. Note that both individuals had a poor cortisol response to surgery.

Pituitary surgeons are familiar with low cortisol states in our post-operative Cushing’s disease patients. In these patients we observe the symptoms of Addison’s disease such as anorexia and malaise. In this setting, it is rare to see marked hypotension, though caution is observed and usual anti-hypertensive medications are held. In two non-Cushing’s patients from our series we observed the same clinical manifestations of hypocortisolemia. These two refused cortisol replacement (Figure 4); indeed one quickly regained eucortisolemia. The clinical value of these observations is that catastrophic cardiovascular collapse is not a characteristic of pituitary (secondary) Addison’s and allows for careful assessment rather than empiric treatment (Glowniak and Loriaux, 1997).

In order to evaluate the integrity and reserve of the HPA axis prior to surgical stress, we desire a simple, reliable measurement. Many clinicians advocate the use of adrenocorticotropic hormone (ACTH) stimulation, but it is costly, difficult to run and there is controversy as to the best way to assess the axis (Aardal-Eriksson et al., 1998; Klose et al., 2005; Ausiello et al., 2008; Pereira and Bevan, 2008). We relied on the most simple of tests, the AM cortisol, as a surrogate for the integrity of the HPA axis. The Abbott ARCHITECT™ cortisol assay used in this study has shown a total CV of <10% for serum samples between 3 and 35 μg/dl and a sensitivity of 20% CV in the lower levels (Abbott, 2009). Given the difficulty and expense of more elaborate testing, using the AM cortisol has been a solution in our practice and, based on this series, appears adequate for the purposes of pre-operative planning for pituitary surgery.

Most patients mounted a considerable rise in their AM cortisol (tripled) in response to pituitary surgery. However, this response was varied from individual to individual, likely in part due to the variable response of patients to surgery, pain, and “stress” in general (Figure 3). However, we believe the post-operative AM cortisol is important to document the preservation of the HPA axis after surgery. Furthermore a similar observation was validated by a series of post-operative pituitary patients from Weil and colleagues. They demonstrated a sensitivity (93%) and a positive predictive value (99%) of the post-op cortisol to predict the preservation of the HPA axis in 100 post-operative pituitary surgery patients, compared to delayed cortrosyn stimulation testing, using 15 μg/dl as the cut off value (Marko et al., 2009, 2010).

Our clinical series has shown that empirical use of stress-dose glucocorticoids is not required for routine pituitary tumor surgery. Physicians who choose not to empirically replace cortisol should be able to recognize the signs and symptoms of hypoadrenalism and may use post-op AM cortisol levels as a measure of post-operative preservation of the HPA axis. Though there seems to be no threat of adrenal crisis with selective use of stress-dose glucocorticoids, our series is too small to be dogmatic in our conclusions and physicians should be able to recognize milder forms of adrenal insufficiency that present as general malaise, appetite loss, and fever. We reserve the right to treat for adrenal crises, should the need arise, but that it is a relatively rare event and, as in our experience with post-operative Cushing’s patients, mild enough to be recognized and treated without catastrophic consequences. The policy presented here appears safe, since there were no apparent adverse events related to lack of empiric coverage; these data re-enforced our larger clinical experience of normal cortisol response in hundreds of pituitary patients over the last 10 years.

## Conflict of Interest Statement

The authors declare that the research was conducted in the absence of any commercial or financial relationships that could be construed as a potential conflict of interest.

## Abbreviations

CSF, cerebrospinal fluid; DI, diabetes insipidus; HPA, hypothalamus pituitary adrenal; μg, micrograms.
